# Potential Applications of Digital Technology in Assessment, Treatment, and Self-help for Hallucinations

**DOI:** 10.1093/schbul/sby103

**Published:** 2019-02-01

**Authors:** Neil Thomas, Josef J Bless, Ben Alderson-Day, Imogen H Bell, Matteo Cella, Tom Craig, Philippe Delespaul, Kenneth Hugdahl, Julien Laloyaux, Frank Larøi, Tania M Lincoln, Björn Schlier, Prabitha Urwyler, David van den Berg, Renaud Jardri

**Affiliations:** 1 Centre for Mental Health, Swinburne University of Technology, Melbourne, Australia; 2 Voices Clinic, Monash Alfred Psychiatry Research Centre, Alfred Hospital and Monash University Central Clinical School, Melbourne, Australia; 3 Department of Biological and Medical Psychology, University of Bergen, Bergen, Norway; 4 NORMENT—Norwegian Center of Excellence for Mental Disorders Research, University of Oslo, Oslo, Norway; 5 Department of Psychology, Durham University, Durham, UK; 6 Department of Psychology, Institute of Psychiatry, Psychology & Neuroscience, King’s College London, London, UK; 7 Psychosis Early Intervention, South London and Maudsley NHS Trust, London, UK; 8 Health Service and Population Research Department, Institute of Psychiatry, Psychology & Neuroscience, King’s College London, London, UK; 9 Department of Psychiatry and Neuropsychology, School for Mental Health and Neurosciences, University of Maastricht, Maastricht, The Netherlands; 10 Mondriaan Mental Health Trust, Heerlen, The Netherlands; 11 Division of Psychiatry, Haukeland University Hospital, Bergen, Norway; 12 Psychology and Neuroscience of Cognition Research Unit, University of Liège, Liège, Belgium; 13 Department of Clinical Psychology and Psychotherapy, Institute of Psychology, Universität Hamburg, Hamburg, Germany; 14 Gerontechnology and Rehabilitation Group, ARTORG Center for Biomedical Engineering, University of Bern, Bern, Switzerland; 15 Institute of Neuroscience, Newcastle University, Newcastle-Upon-Tyne, UK; 16 Research and Innovation, Parnassia Psychiatric Institute, The Hague, The Netherlands; 17 PsyCHIC Team, SCALab CNRS UMR-9193, Lille University, Lille, France; 18 CURE Platform, CHU Lille, Fontan Hospital, Lille, France

**Keywords:** digital mental health, mHealth, Internet, smartphones, hearing voices, schizophrenia

## Abstract

The field of digital mental health is rapidly expanding with digital tools being used in assessment, intervention, and supporting self-help. The application of digital mental health to hallucinations is, however, at a very early stage. This report from a working group of the International Consortium on Hallucinations Research considers particular synergies between the phenomenon of hallucinations and digital tools that are being developed. Highlighted uses include monitoring and managing intermittently occurring hallucinations in daily life; therapeutic applications of audio and video media including virtual and augmented reality; targeting verbal aspects of hallucinations; and using avatars to represent hallucinatory voices. Although there is a well-established Internet-based peer support network, digital resources for hallucinations have yet to be implemented in routine practice. Implementation may benefit from identifying how to market resources to the broad range of populations who experience hallucinations and identifying sustainable funding models. It is envisaged that digital tools will contribute to improved self-management and service provision for people experiencing hallucinations.

Hallucinations are real-seeming perceptual experiences that arise without corresponding sensory input, often in the form of hearing voices or seeing visions.^1^ Although many people experience hallucinations without requiring professional treatment or service delivery,^2,3^ some of these experiences can be persisting and lead to significant distress and disability. Clinical populations in which they may be a focus of assessment or treatment span diagnoses of schizophrenia-related, mood, dissociative, trauma-related, and neurological disorders, as well as a range of infectious, autoimmune, metabolic, and genetic diseases.^4^ Using antipsychotic medication to treat hallucinations—the first line treatment in psychotic disorders^5^—has variable effectiveness, with many continuing to experience hallucinations.^6^ Additionally, medication is often not taken as prescribed,^7^ bears the risk of severe adverse effects,^8^ and there is a growing concern over the cumulative health effects of long-term use.^9^ This underlines the necessity to develop safer treatments and ones that are more readily accepted by patients. The most common alternative forms of intervention include individual and group psychological therapies^10^ and peer support,^11^ both aiming to promote better adaptation to the experience, and reduce associated distress and impact on functioning.^10^ Intervention research for hallucinations has primarily focused on the experience of hearing voices in the context of schizophrenia spectrum diagnoses,^8^ where therapies, mostly within a cognitive-behavioral therapy (CBT) tradition, have included elements such as enhancing coping and self-management,^12,13^ cognitive restructuring of beliefs about voices as powerful others,^14^ exploring alternative ways of interacting with voices,^15^ targeting memories associated with distressing voice content,^16,17^ and acceptance- and mindfulness-based approaches.^18,19^

While psychological interventions are a recommended part of practice, their effects have been modest across approaches,^20–23^ although targeted approaches for hallucinations seem to be showing stronger effects.^24^ A further challenge is that psychological therapy has proven difficult to implement on a widespread basis.^25^ Limiters to the reach of psychological therapies include lengthy training requirements for practitioners, and a lack of prioritization within models of service delivery used where persons access help.^26^ A potential means of progress in both effectiveness and in reach is to look at approaches that extend beyond traditional models of expert-delivered consultation room psychotherapy.^25^ In other contexts, digital technologies have been successfully applied to mental health problems. These have included self-management programs, assessment tools, telehealth, mobile apps, serious games, virtual reality, and wearable sensors.^27–31^ These approaches have been accompanied by promising outcomes and can help increase rates of delivery both via making self-guided programs directly accessible and via supporting empirically supported treatment delivery by health practitioners.^27,32^ Additionally, use of mobile apps (accessible during day-to-day life), and virtual reality may help to increase the potency, dose, and/or tolerability of what can be achieved using traditional therapies.^29,31,33^

Although there have been a number of reviews considering the development of interventions for severe mental illness,^34–40^ hallucinations are encountered in a range of populations and possess a distinct phenomenology that may present specific use cases for digital technologies that are becoming increasingly accessible. This article presents a review from an international working group of researchers from the International Consortium on Hallucinations Research (ICHR),^41–44^ currently involved in applying digital technologies to the experience of hallucinations. The working group was formed by approaching some of the investigators of projects to develop hallucination-specific digital applications, identified via an initial search of published articles, plus other researchers in the consortium involved in developing new applications or paradigms. The aim of the review was to consider the current, emerging, and future potential applications of digital technologies to aid assessment, treatment, and/or self-management of hallucinatory experiences as a specific target. We considered this in terms of identifying specific ways technology could be used to target hallucinations and the contexts in which digital applications could be implemented with this population. The scope was restricted to hallucinations, rather than a broader spectrum of experiences such as psychosis. Hallucinatory experiences were considered across different sensory modalities, and different diagnoses/populations.

Existing published literature was identified via search of multiple indexing databases using relevant search terms, plus manual search of reference lists and citing articles. Consideration of this literature was combined with discussion among the working group via video conferencing, email discussion, and at the fourth meeting of the ICHR in Lille, November 2017.^44^ This article presents a narrative review which synthesizes the existing literature with themes from working group discussion of future developments.

## Digital Applications to Voices Reported in the Literature to Date

Although there are examples of using mobile apps to collect research data on hallucinations,^45–47^ and hallucination simulations used in medical/health education,^48,49^ there are relatively few reports on clinical or self-help uses of digital applications for hallucinations. Of those that have been reported, the majority reference hallucinations as a (small) aspect of a self-guided web program or mobile app for persons with schizophrenia spectrum disorders.^50–58^ This is a population that has not seen as heavy development in digital mental health technology, despite increasing rates of internet and smartphone access.^59–62^ Digital programs for persons with schizophrenia spectrum disorders have primarily targeted symptom management, improving functioning, and relapse prevention. Hence, the material on hallucinations has been provided in this context, for example, being included as an item on a self-monitoring system for relapse prevention,^52,55–57^ or as a component of digital materials on symptom self-management.^50,53,54,58^ Some applications have utilized branching logic from responses entered by users to provide tailored prompts according to symptoms being reported, with material on coping with hallucinations being provided only when relevant.^50–52,54^ Findings of the available, mainly small-scale, studies fit with a broader literature that suggests that digital programs are a feasible and acceptable medium for intervention delivery among persons with mental health problems, including those with disabling mental health problems such as schizophrenia spectrum disorders.^34–40^ However, with hallucinations representing only a small component of these interventions, it is difficult to derive clear conclusions about their acceptability and usefulness for this specific target.

In considering digital applications that were developed specifically for hallucinatory experiences, there were 3 notable fully developed clinical/self-help applications reported on in the published literature. The first, *Coping with Voices*,^63,64^ is an online self-management program developed for people hearing voices with a psychotic disorder diagnosis. Adopting a widely used approach in online programs for mental health self-management, this comprised a web-based course of 10 CBT-based modules incorporating material on self-monitoring, coping enhancement, cognitive therapy skills, and relapse prevention. Delivery methods included the use of text, video, audio, and interactive exercises. Results of 2 small studies demonstrate feasibility of self-paced use, with approximately 80% of participants completing the intervention (when delivered on-site in a mental health service), and high rates of satisfaction, but effects on outcome are unclear.^63,64^

The second is AVATAR therapy, initially described in a pilot study in 2013,^65^ and recently evaluated in a randomized controlled trial (RCT) compared with a supportive counseling control condition.^66,67^ In this approach, a combination of digital image and speech modulation software is used to generate an animated avatar. This avatar is voiced by the therapist but transformed by the software to resemble the tone and characteristics of a chosen distressing voice. During therapy sessions, the therapist controls the avatar from another room to facilitate a trialogue between participant, avatar and therapist, supporting the participant gaining power over the initially powerful and threatening voice which becomes more conciliatory over time. The RCT confirmed the positive outcomes of the pilot study with significant reductions in the frequency, associated distress, omnipotence, and power of voices compared with that achieved by the supportive counseling control condition at 3 months. Therapy gains were maintained at 6-month follow-up, although they were no longer significantly different from the control condition.^67^ A further study by an independent group has conducted AVATAR therapy in immersive virtual reality and found this to be feasible with large estimated effects.^68^

Third, Demeulemeester et al^69^ reported on the Multisensory HAllucinations Scale for Children (MHASC), an assessment tool to help children discuss their hallucinatory experiences in all modalities (http://mhasc.eu/). This app uses common videogame-based aesthetics to increase engagement and motivation of children during the assessment, notably using a playful interface and developmentally appropriate language. A validation study is ongoing, and the app is being translated into several languages.

Overall, these reports in the literature demonstrate the feasibility of using digital tools to support hallucinations assessment, as a form of self-guided intervention, and as a component of in-person therapy. However, the modest literature available also shows this work is at an early stage: most innovation in this space includes applications that are either still in development or currently being tested for their efficacy.

## Applying Digital Technology to Specific Characteristics of Hallucinatory Experiences

Of the early applications described above, AVATAR Therapy is notable in harnessing digital technology to target a unique characteristic of hallucinations. In considering how digital applications may further progress by targeting specific aspects of hallucinatory experiences, we considered 4 key synergies that appear promising foci for development, listed in table 1. We discuss these in turn.

**Table 1.** Potential Synergies Between Digital Technologies and Hallucinations

Typical Characteristic of Hallucinations	Corresponding Digital Technologies
Occurring intermittently within day-to-day environment	Ecological momentary assessment in monitoring; ecological momentary intervention in self-managing
Perceptual phenomena	Audio and/or visual media; virtual and mixed reality
Verbal content	Digital speech-based stimuli
Interpersonal content	Avatar representations of voices and self; autonomous interactive agents using natural language processing

### Monitoring and Managing Hallucinations in Daily Life

The main persons in need of intervention are those whose hallucinatory experiences are frequent and distressing. In this group, hallucinations are usually experienced intermittently throughout the day, and although some experience hallucinations on an almost continual basis, it is typical for some variability to occur.^70^ Digital devices such as smartphones provide a vehicle for bridging between the consultation room, where voices are often not present, to situations in which they occur.

This presents opportunities from the therapeutic application of methods based on *ecological momentary assessment* (EMA) research.^71^ Also known as the *experience sampling method*,^72^ EMA involves prompts being delivered at random intervals throughout the day encouraging people to provide current information so that data can be recorded in the moment (eg, during or shortly after experiencing hallucinations) rather than via retrospective recall. One benefit of this is in capturing more ecologically valid data, which facilitates, for example, more accurate symptom monitoring and outcome measurement.^73^ A further application is in determining temporal relations between variables, providing individual-level data on the relationships between contextual variables and experiences. EMA research has been successful in determining relationships between hallucinations and levels of meaningful activity,^45^ time of day,^74^ emotional state,^45^ autonomic regulation,^47^ and worry/rumination.^75^ Mobile devices allow contextual variables to be monitored via prompted user entry (eg, worry), and/or by passive capture of behavioral (eg, location history, activity) and physiological (eg, autonomic arousal, heart rate variability) data to predict hallucination on- and offset. Moreover, there is potential for this method to be applied to develop an individualized functional analysis of the occurrence of hallucination to inform individual management.^76,77^

Similarly, there is potential for EMA prompts to be used to support the regular implementation of self-management strategies, termed *ecological momentary intervention* (EMI).^29,76–78^ This may include simple reminders of coping strategies delivered during day-to-day life, or coping statements programmed to provide different responses according to user indications of the presence/absence and/or intensity of hallucinations. An initial trial of an intervention for hallucinations based on EMA/EMI is underway in Melbourne.^77^ Further work regarding these uses of EMA/EMI includes developing meaningful strategies for data analysis and visualization on an individual basis;^79^ identifying the most acceptable response schedules; optimizing the thresholds underlying the branching logic from user-responses to prompts with relevant self-help material; contrasting time-limited focused interventions with programs designed for maintenance; and considering the need for therapist support in using effectively.

In the longer run, there may be potential of using contextual and physiological data that have been found to be linked to hallucination^46,47^ to prompt interventions. This will require a substantially improved understanding of contextual and physiological antecedents of hallucinations. A particular area for future growth in this regard is likely to be data capture from the use of nonobtrusive environmental and wearable sensors. Wearable technology refers to sensors or devices that can be regularly worn by an individual and collect (continuous) data for inferring the person’s behavior, physiology, and the environment. While hallucinations are inherently private experiences, these data provide potential opportunities for observable indices that can be associated with their presence. (heart rate, blood pressure galvanic skin response) can physiological (eg, heart rate, blood pressure, and galvanic skin response to assess autonomic stress) and behavioural (eg, movement levels) parameters can be linked to variation in hallucination severity, both in daily living or in a virtual reality setup. There is also potential to index visual exploration behaviour through virtual reality headsets whilst in virtual environments.^80^ Laboratory-based studies have linked the occurrence of positive symptoms with observable autonomic system abnormalities,^81–83^ and heart rate variability measured with wearable devices has been associated with reported positive symptoms in general^84^ and hallucinations specifically.^47^ These early findings suggest that unobtrusive collection of data through wearable devices may be used to develop parameters to model the presence of hallucinations. Combining networks such as the ICHR to collect vast datasets coupling sensor-based data with the subjective account of the person experiencing hallucinations with use of machine learning may improve precision with which we can predict the occurrence of these events, in turn, supporting self-management tools.^85,86^ However, while there has been an emphasis on the advantages of, however, while there is new potential in applying statistical analysis to vast datasets, it is important that experts, including people who experience hallucinations, are involved in developing models of how the information collected by passive monitoring techniques can inform prevention, treatment, service delivery and self-management. As with other areas, it is imperative that developers and providers connect with users and manage negative consequences such as concerns about private experiences being monitored by others, and myriad ethical issues.^87^

### Using Digital Audio and Video With the Perceptual Qualities of Hallucinations

Hallucinations, by definition, are perceptual phenomena, most often auditory or visual. This makes them potentially suited to applying audio and visual digital media. First, audio media may have some applications in providing alternative sensory stimulation for persons with auditory hallucinations. Experimental evidence supports that meaningful auditory stimulation may reduce hallucinatory experiences,^88^ and auditory stimulation such as listening to music through headphones has long been used as both a naturally arising coping strategy and simple self-management intervention.^12^ There is potential for smartphone integration to provide a resource tailored for day-to-day coping. For example, the Bergen fMRI Group has developed a smartphone application, which uses conflicting auditory stimuli (presented via headphones) to train patients to focus on sounds other than their voices.^89^ There is evidence that training with this task reduces voice duration^90^ and promotes alternative coping strategies.^91^

Second, digital technologies can be used to generate hallucination-like stimuli. Multiple hearing voices simulations exist in the form of simple auditory recordings of an actor simulating common voice content, typically played through headphones.^48^ To date, their use has primarily been in medical and public education about the experience (where research findings in fact caution that they may unintentionally result in increased stigma).^48^ In considering the therapeutic usage of such simulations, a possible role is as material for clients practicing the learning and application of different coping methods. Use may, in turn, support habituation to threatening content, increase perceived control over experiencing hallucinations, and reduce associated distress. However, given the individual nature of voices, content aligned with the person’s own experiences may be achieved by recording examples representative of the person’s own voice content, as has been used in training in mindfulness applied to voices.^92^

The more challenging task of recreating visual hallucinations has also seen some development in relation to simple visual hallucinations. An example from a nonhealth literature involved developing a tablet-computer augmented reality simulation of palinopsia, a visual disturbance common in neurological disorders involving persisting afterimages of objects in the visual field.^93^ This “Halluciphone” application was co-developed by a musician as a performance arts work, but with a view to broader use as a communicative tool. Anecdotal reports in the article of persons with palinopsia suggested that it had value in helping persons with this experience understand it and communicate it to others.^93^ Another visual hallucination simulation that has been described is the “Hallucination Machine” which uses panoramic virtual reality to present distortions resembling those experienced during use of hallucinogens,^94^ but its clinical applications have not been considered. The development of virtual or augmented reality representations of complex hallucinations is more difficult due to technological limitations in the extent of realism that can be achieved particularly with hallucinations in human or animal form. However, developments in virtual reality for paranoia^95^ as well as findings of the AVATAR trial suggest that computerized avatars can provide a therapeutically useful representation of others in the environment. There are also observations that people prone to visual hallucinations (in Parkinson’s disease) can experience them within immersive virtual environments.^96^

New technologies also represent a way of making laboratory tasks accessible in clinical practice. For example, the auditory signal detection paradigm^97^ represents a means of eliciting hallucination-like experiences and measuring hallucination-proneness. In this paradigm, participants listen to recordings of ambiguous noise containing barely audible embedded voices (or not) and are asked to indicate if any words/voices were detected. Most studies have used white noise as ambiguous material but other types of noise may be more effective for eliciting hallucinations (eg, pink noise, people babbling). A meta-analysis^98^ showed that both clinical and nonclinical individuals with hallucinations tend to perceive more words/voices that are not actually in the noise (ie, false alarms) than people with a lower proneness toward hallucinations. This has potential applications both as an objective and stigma-free assessment of hallucination proneness, and as a future clinical tool for training attentional and cognitive responses.

### Targeting Verbal Aspects of Auditory Hallucinations

Hallucinatory experiences, typically in the form of voices, are seen in many models to reflect the involvement of cognitive processes associated with inner speech.^99^ This raises the prospect of targeting characteristics of inner speech to initiate therapeutic change, such as by diverting speech-related processing to other tasks, or disengaging from inner dialogue when this is unhelpful. At a basic level, this might be done by using a smartphone audio player to present coping exercises in verbal form, or exercises that promote disengaging from inner verbal processes (eg, mindfulness exercises designed for in-the-moment coping with voices^81^). Similarly, *Temstem*,^73^ is an app that contains 2 language games and aims to enhance control over auditory verbal hallucinations (AVH) by activating the language production areas in the brain, which has been found to suppress AVH. The app also aims to enhance self-esteem by providing feedback that competes with the negative self-schemas that are activated by the AVH. Temstem contains a dual task function that is based on the finding that taxing working memory during recall of negative visual or auditory imagery, reduces the vividness and emotionality of imagery and the frequency of relieving symptoms.^100^ Inner speech itself also appears to be amenable to being captured by EMA,^101^ which may present possibilities for integrating with EMA/EMI-based approaches described above.

### Using Digital Representations of Auditory Hallucinations as Perceived Others

Hallucinations in the form of voices are typically experienced as being articulated by another person, with the hearer responding to them as if they are communicating with sentient others, either verbally or mentally.^102^ Indeed, it has been argued that the perception of the presence of individualized “agents” may be a fundamental aspect of the experience.^103^ The AVATAR trial addressed this by creating a “concrete” avatar representation of this experience to facilitate cognitive restructuring and practicing role plays of different ways of responding akin to those used in nondigitally supported therapies.^14,15^ One next step with this may include determining whether the technological component of this intervention creates a bigger impact than the use of role plays per se, which themselves show preliminary evidence of being effective.^15^

This apparent success in being able to use computer-generated avatars to represent voices raises potential applications beyond their demonstrated feasibility in patient–therapist role plays. One possible direction is using avatars within a self-directed program, for example, by the client being able to generate an avatar using their own prerecorded examples of voice content, to support habituation and defusion from content. Potentially, further development may utilize natural language processing used in voice assistant and chatbot technology in order to tailor responses to how people interact with their voices verbally to practice different responses. The use of avatars may additionally provide means to manipulate the avatar as a virtual voice representation (eg, turning down the volume, reducing its size) which might be used in reducing its sense of dominance over the hearer. This method is already used via metaphors and imagery in other therapies,^17,92^ but may become more accessible using a digital tool thanks to elements of gamification.

This potential for avatars to represent voices raises the question of whether effects might be enhanced or even attenuated using immersive virtual reality.^104^ Use of virtual reality could produce a more vivid and powerful visual experience or allow avatar representations of other entities to be incorporated such as representations of the therapist, of trusted others, or benevolent voices as allies in supporting the person responding to critical or threatening voices. In the future, augmented reality could be used to bring an avatar representation of voices to life superimposed onto the person’s environment, either via a virtual reality headset, or live camera feed on a smartphone or tablet computer. However, these opportunities are balanced against the core purpose of the AVATAR trial in creating a meaningful dialogue between avatar and participant, so too great a presence of other entities in the virtual environment might dilute the impact of the dialogue. In addition, next-generation virtual reality applications could include virtual scenarios with automatic adjustment of difficulty to error rates or arousal, which may render self-management using (home-based) virtual reality applications feasible.

## Peer Support

In addition to using digital technology to target these phenomenological characteristics of hallucinations, it is notable that this is an experience associated with a particularly strong peer network. The international Hearing Voices Network (HVN) has been one of the strongest components of the consumer movement in mental health, advocating for the needs and rights of persons who identify with the experience of hearing voices, and coordinating peer support, primarily in the form of local hearing voices groups.^11^ The HVN has been an early adopter of the internet as a means of promoting peer support, actively using bulletin boards and email mailing lists among voice-hearers since the early days of the Internet, extending to the formation of Facebook groups, and the intervoiceonline.org and local websites.^105^ In addition to using these asynchronous mediums of communication to connect voice-hearers, the HVN has innovated in organizing virtual peer support hearing voices groups using text-based chat or video conferencing for persons unable to attend in-person groups.^106^ There has yet to be systematic research on this. A key question regards chat vs video formats, since each has potential advantages: chat requiring less internet speed and data, and potentially allowing for anonymity or participation without needing to commit to active interaction; and video conferencing potentially allowing interaction that most closely resembles an in-person group. Peer-to-peer support has been highlighted as a key application of digital health,^107^ and this appears to be an area of growth. Peer support networks also provide a means of engaging people in other resources.^108^

A further example of digital technology promoting learning from shared lived experience has been the use of multimedia featuring peers communicating stories. A particularly influential lived experience video has been the TED talk by Eleanor Longden, that has been widely viewed (over 4 million views at the end of 2017).^109^ Video streaming material is often used for mental health information among persons with severe mental illness,^62^ and is being increasingly used in online programs, including as the main means of communicating content for people with persisting psychosis.^110,111^ A potentially important direction for ongoing research in this area is to examine the impact of peer video vs other types of communication, and potential processes involved in this and the impact they have on persons with shared experiences.

## Translation into Routine Provision

How much can these digital technologies translate into routine provision? It is notable that trials of Coping with Voices, Avatar and MHASC have all primarily been developed with implementation by in-person services in mind, relying upon integration with services and significant clinician support. An implementation study that examined the uptake of a suite of digital resources in persons with schizophrenia-related disorders following discharge for an acute psychotic episode reported 85% used the EMI-based FOCUS app^51^ which includes a module on hallucinations, and 59% used either Coping with Voices or a similar self-management course for paranoia.^52^ This suggests that when there is an implementation initiative, integration of digital tools leads to uptake, at least in services for severe mental illness.

However, it has yet to be tested whether making tools available via the internet can lead to direct client uptake in this particular population. In spite of the potential for reach of the internet it is noteworthy that, at the time of writing, there are virtually no dedicated publicly available resources for hallucinations, outside websites associated with the HVN. To address this gap, Durham University’s Hearing the Voice project recently began work on *Integrated Voices*, a website that will bring together knowledge on voice-hearing from experts-by-experience, clinicians, academics, and other stakeholders. This process has started with an extensive consultation phase^112^ that has identified living with voices (in terms of day-to-day management of experiences, what to tell work, coping at school) as key area where people would like more support information. Large sections of the site will be also co-produced with voice-hearers. The site will launch in early 2019.

Providing information and tools that can be directly accessed presents an opportunity to break away from traditional service-based boundaries such as clinical/nonclinical, psychiatry/neurology and child/adult, enabling resources for hallucinations to be delivered across diagnostic boundaries. However, it is untested whether universal resources are feasible in meeting needs of different populations. Among mental health service users, hallucinations involving hearing voices are common, so programs targeting auditory, verbal and related interpersonal characteristics may be of value. On the other hand, in neurological populations visual hallucinations are more typical, so developments in virtual reality may be of greater value. Considering the logistic challenges of these innovative technologies, psychoeducation, EMA/EMI and peer support may have more universal applicability. In considering potential user engagement with “one size fits all” solutions, it is unknown how much people would identify as part of the same group, and how heterogeneous preferences are for use of language (eg, “hearing voices” vs “hallucinations”), and how experiences are framed (eg, as human phenomena vs symptoms of brain dysfunction). Sensory modalities and shared experiences (eg, commanding vs critical voices; trauma-related vs nontrauma related experiences)^113,114^ may present alternative ways of organizing content. Ultimately, it is likely that persons with hallucinatory experiences share a combination of specific needs and commonalities, and this emphasizes the importance of co-design and input from representatives across the spectrum of these experiences in considering implementation in practice.

A further consideration in implementation is funding. There are significant costs involved when developing digital interventions which health care providers and researchers need to consider carefully. Upfront development costs are significant, and software requires hosting, maintaining, and servicing. Content needs updating to retain engagement, and to adapt for different languages and cultures. This requires that developers consider ongoing access to funding allowing for the necessary technology maintenance and update. The necessary innovation cycle of internet applications is at odds with how methods are developed and evaluated in evidence-based health care. Health care interventions are typically evaluated with a rigorous series of studies evaluating initially the acceptability and feasibility of a new intervention, then its efficacy and at last its effectiveness.^115^ These intervention development stages are costly and time-consuming: it is often the case that by the time a digital intervention has been developed in its original form and evaluated in an RCT, both the hardware and the software will be outdated. This is particularly important when the overall market for health apps is in such high growth that it is already flooded with apps featuring content developed without significant input of relevant clinical, consumer, or research expertise.

It is often advocated that the evaluation of digital interventions should follow a more dynamic pathway.^30^ Indeed, it is questionable whether finite research time is best used separately developing and trialing competing apps with overlapping functions as if they were distinct new treatments. Our efforts may be more productive in examining the various ways in which technology can be employed with hallucinations, to develop rigor around how digital technology can be used with this experience, and to inform further innovation.

## Conclusion

The application of digital technology to hallucinatory experiences is at a very early stage, but emergent technologies offer significant potential to target the perceptual, verbal, and person-like characteristics of hallucination, and develop greater sophistication in using technology for mapping this experience on an individual level. We envisage this growth will provide more accessible resources that support better treatment delivery and independent self-management.

## Funding

This work was supported in part by an Australian Government Research Training Program Scholarship (IHB), a Wellcome Trust Award (WT108720 to BAD), and Eurostars (grant number 11010 to BS).
